# Nurses’ Experiences Using AI in Clinical Practice: Systematic Review

**DOI:** 10.2196/91238

**Published:** 2026-06-25

**Authors:** Ashley J S Scott, Qimeng Zhao, Jo-Fan Pan, Benjamin C Brown, Dawn Dowding

**Affiliations:** 1 Division of Nursing, Midwifery and Social Work, School of Health Sciences, Faculty of Biology, Medicine and Health, University of Manchester, Oxford Road, Manchester, England, M13 9PT, United Kingdom, +44 161 306 9966; 2 School of Nursing, Faculty of Applied Sciences, University of British Columbia, Vancouver, BC, Canada; 3 Division of Population Health, Health Services Research and Primary Care, School of Health Sciences, Faculty of Biology, Medicine and Health, Manchester, England, United Kingdom

**Keywords:** artificial intelligence, nursing, clinical practice, user experience, decision support systems, technology acceptance model, implementation, systematic review, PRISMA

## Abstract

**Background:**

Artificial intelligence (AI) tools are increasingly used in clinical settings, yet most syntheses focus on nurses’ attitudes or readiness rather than experiences after direct use in practice.

**Objective:**

This study aimed to synthesize registered nurses’ experiences of using AI in clinical practice and to identify perceived benefits, barriers, and implementation implications.

**Methods:**

We conducted a systematic literature review of empirical studies reporting nurses’ experiences of AI use in clinical settings. Searches were performed in CINAHL, Embase, MEDLINE, PsycINFO, and PubMed (last search: September 13, 2023). Moreover, 2 reviewers (AJSS and QZ) independently screened titles, abstracts, and full texts and appraised included studies using the Mixed Methods Appraisal Tool. We used thematic synthesis with a primarily deductive framework based on the Technology Acceptance Model 2, with the addition of facilitating conditions from the unified theory of acceptance and use of technology.

**Results:**

In total, 20 studies met the inclusion criteria. Perceived usefulness and facilitating conditions were most frequently reported; nurses described AI as supporting decision-making, workflow efficiency, and confidence when implementation included adequate training, interoperability, and technical infrastructure. Ease of use was closely tied to interface design and training. Job relevance and output quality were rated more positively when nurses described AI as aligning with nursing tasks and as producing interpretable, reliable outputs. Common barriers included usability issues, limited integration into workflows and electronic systems, privacy and trust concerns, and inconsistent or poorly contextualized outputs. Across studies, nurses often described adoption as conditional on organizational readiness and meaningful involvement of nurses in design and implementation.

**Conclusions:**

Nurses’ accounts suggest that AI may augment clinical work, but the perceived benefits reported in the included literature were contingent on workflow alignment, usable interfaces, training, and supportive infrastructure. These findings suggest potential value in involving nurses in the co-design and iterative refinement of AI tools, grounded in the consistent evidence that facilitating conditions, such as training, interoperability, and organizational readiness, are the primary determinants of whether AI is experienced positively. Prospective evaluation using nursing-relevant outcomes, such as usability, workflow integration, and trust, is needed to move beyond post hoc experiential accounts and establish what implementation conditions reliably produce benefit.

## Introduction

The integration of artificial intelligence (AI) into health care has transformed clinical practice, significantly impacting the roles and responsibilities of registered nurses (RNs). As the largest group of health professionals, RNs are at the forefront of these technological shifts and have historically been an afterthought in technology development [1]. AI holds significant potential to enhance nursing practice in administration, clinical practice, policy, and research, but it remains an area of ongoing exploration [2]. For example, natural language processing systems are increasingly used to assist with clinical documentation, decision support algorithms enhance diagnostic reasoning, and in nursing administration, AI has been applied to workload prediction and staff scheduling, while in research, it supports pattern recognition in large datasets [3].

AI’s emergence in health care has altered nursing roles, workflows, and the nurse-patient relationship [4,5]. Technologies like predictive analytics, virtual health care assistants, and robotics are influencing nursing practice in substantive ways: (1) predictive analytics supports early identification of deteriorating patients, enabling more proactive clinical intervention; (2) virtual health care assistants facilitate patient education and remote monitoring; and (3) robotic technologies are increasingly used for medication dispensing and patient mobility support, reshaping how physical tasks are distributed [6-8]. While these AI-driven technologies offer improvements in health care delivery, they also challenge the maintenance of person-centered, compassionate care [2]. AI already contributes significantly to decision-making support and skill augmentation within clinical practice [9]. Nurses must proactively ensure AI technologies align with nursing’s core values, maintaining ethical and compassionate care [10].

The integration of AI into nursing practice is accelerating, and existing systematic reviews predominantly explore general perceptions and theoretical readiness or educational readiness among nurses [11,12]. These studies offer valuable insight into anticipated benefits and challenges, yet conclusions are derived from nurses who have not directly interacted with AI in clinical settings. For example, 1 recent review synthesized qualitative studies focused on nurses’ perceptions without requiring previous AI use [12]. Several recent reviews have examined AI specifically within nursing education contexts; one explored AI use in nursing training [13], while another synthesized empirical evidence on AI implementation in hospital settings using a strengths, weaknesses, opportunities, and threats framework [14], and another mapped AI applications, such as generative tools and virtual patient simulators, in university-level nursing programs [15]. All 3 address AI as a pedagogical resource for nursing students and trainees, rather than clinical practice. There remains a lack of synthesis of studies capturing nurses’ attitudes after engaging directly with AI tools.

Several theoretical frameworks can be used to understand individuals’ acceptance and use of technology, such as AI. The Technology Acceptance Model 2 (TAM2) is among the most extensively validated, proposing that perceived usefulness, perceived ease of use, and social and cognitive influences shape technology adoption [16]. TAM2 was developed in general organizational technology contexts, and we acknowledge that it may underweight dimensions that are particularly salient in nursing, including professional judgment, the nurse-patient relationship, ethical responsibility, accountability for patient outcomes, and the emotional and moral dimensions of clinical work. We selected TAM2 nonetheless because our review question concerned nurses’ acceptance-relevant experiences after direct AI use, where TAM2’s validated constructs provide an established and transparent analytic vocabulary. To address TAM2’s primary acknowledged limitation, its limited treatment of organizational and infrastructural context, we incorporated the Facilitating Conditions construct from the unified theory of acceptance and use of technology (UTAUT) [17], which captures the technical, training, and resource conditions that nurses’ accounts suggested were central to their experiences. We did not adopt the full UTAUT to avoid construct duplication with TAM2 (the implications of this deductive choice for what the synthesis could and could not surface are discussed in the Limitations section). This review explores the experiences of RNs navigating modern health care complexities, with a focus on AI’s impact on nursing roles, the challenges in adapting to AI technologies, and how these changes are perceived to influence patient care using TAM2 and UTAUT’s Facilitating Conditions as the analytical framework.

The research question guiding this systematic review is “What are the experiences of nurses using AI in their clinical work?” The objectives are to (1) develop a better understanding of how nurses interact with AI in clinical practice, and (2) provide insights into nurses’ perceptions of AI’s impact on efficiency, satisfaction, and nurse-patient relationships.

## Methods

### Overview

The primary objective of this systematic review is to understand the experiences of nurses using AI in clinical practice.

A systematic review methodology was selected because the research question required a comprehensive, transparent, and reproducible synthesis of empirical evidence from multiple studies. This approach is appropriate for identifying and integrating findings across a body of literature to address a focused question about a specific population’s experiences, and is consistent with established guidance for evidence synthesis in health professions research [18].

### Protocol and Registration

The study protocol was registered with PROSPERO (CRD42022308051) [19]. The manuscript title has been refined from the registered title for clarity; the review question, eligibility criteria, and methods are consistent with the registered protocol.

### Ethical Considerations

Ethics approval was not required because this study synthesizes data from previously published literature and did not involve collection of identifiable participant data.

### Eligibility Criteria

Eligibility was determined using predefined inclusion and exclusion criteria (Textbox 1).

Textbox 1. **Eligibility criteria.**

**Inclusion criteria**

- Empirical research published in English: to ensure consistency and enable thorough analysis, only studies published in English were included.
- Peer-reviewed journal articles: studies published in peer-reviewed journals were prioritized for their methodological rigor and reliability.
- Studies where registered nurses describe their experiences of using artificial intelligence (AI) in clinical practice: the review focused on direct, real-world experiences of registered nurses with AI technologies. For inclusion, studies were required to report on nurses’ experiences or perceptions arising from actual use of or interaction with an AI tool in a clinical setting. Studies reporting only on anticipated, hypothetical, or theoretical attitudes toward AI, without confirmed direct use, were excluded.
- Non–peer-reviewed sources (such as poster presentations and conference abstracts): these would have been considered only if they contained unique empirical data not yet published in a peer-reviewed format and demonstrated methodological rigor as assessed using the Mixed Methods Appraisal Tool (MMAT) criteria applied to all included studies.

**Exclusion criteria**

- Studies published in languages other than English: non-English language publications were excluded due to language barriers affecting interpretation.
- Studies reporting the experiences of nurses using technology not underpinned by AI: studies reporting on technologies that were not underpinned by AI were excluded.
- Studies reporting the experiences of nurses working in non-clinical settings: research focusing on administrative, academic, or educational settings outside of clinical practice were excluded.
- Studies related to health care professionals (eg, midwives and doctors): studies were excluded unless they specifically reported data related to registered nurses.
- Duplicate reports of the same study: the most comprehensive versions of duplicate studies were included.

### Information Sources

A comprehensive literature search was conducted on September 13, 2023 across 5 electronic databases—PubMed, Embase, MEDLINE, CINAHL, and PsycInfo.

### Search Strategy

A search strategy was developed and refined through repeated testing and adjustment, using both keywords and controlled vocabulary tailored to each database. Concept blocks, illustrated in Textbox 2, provided the structure for the search, with keywords and controlled vocabulary terms merged using Boolean logic (AND/OR) to ensure comprehensiveness. No date restrictions were applied, allowing for the inclusion of all eligible studies up to the date of the search. Searches were not restricted by language, but inclusion was limited to studies published in English. Reference lists of identified articles were also reviewed, but no additional studies met the inclusion criteria. Full search strategies are provided in Multimedia Appendix 1.

Textbox 2. **Search strategy concept blocks.**

**Block 1: Nursing**

- Keywords: Nurse* (Title or Abstract), “Nursing staff” (Title or Abstract), “Nursing professional*” (Title or Abstract), “Registered nurse*” (Title or Abstract)
- CINAHL Headings: Nurses, Nursing Staff

**Block 2: Artificial intelligence**

- Keywords: “Artificial Intelligence” (Title or Abstract), “Machine Learning” (Title or Abstract), “Neural Network*” (Title or Abstract), “Deep Learning” (Title or Abstract), “Computer Vision” (Title or Abstract), “Natural Language Processing” (Title or Abstract)
- CINAHL Headings: Artificial Intelligence, Machine Learning

**Block 3: Experiences or perceptions**

- Keywords: Experience* (Title or Abstract), Perception* (Title or Abstract), View* (Title or Abstract), Attitude* (Title or Abstract), Feedback (Title or Abstract), Opinion* (Title or Abstract)
- CINAHL Headings: Attitude, Perception

### Study Selection

The review was conducted following the Joanna Briggs Institute (JBI) methodology for systematic reviews, using the “JBI Manual for Evidence Synthesis” [20] as the guiding framework. The reporting of this review adheres to PRISMA (Preferred Reporting Items for Systematic Reviews and Meta-Analyses) 2020 guidelines [21]. Study management and screening were managed with Covidence software (Veritas Health Innovation Ltd). Furthermore, 2 reviewers (AJSS and QZ) independently screened titles, abstracts, and full texts, resolving discrepancies through discussion and consensus. In cases where multiple reports of the same study were identified, the most comprehensive version was prioritized, and duplicates were merged accordingly. The screening and selection process are presented in the PRISMA flow diagram in the Results section. The completed PRISMA 2020 checklist is also provided in Checklist 1.

### Data Extraction

Data extraction followed the JBI mixed methods data extraction form following a convergent integrated approach [20]. Where studies included multidisciplinary samples, nurse-specific findings were extracted separately where the primary data permitted. In cases where profession-specific disaggregation was not possible, this was noted explicitly in the data extraction table (Multimedia Appendix 2).

### Quality Appraisal

Study quality was assessed using the Mixed Methods Appraisal Tool (MMAT) [22] to accommodate the diversity of included study designs. This aligned with the convergent integrated approach used in this review [20]. Additionally, 2 reviewers (AJSS and QZ) independently assessed study quality, and any discrepancies were resolved through discussion to reach consensus. To capture the complexity of interpretive and critical understandings of AI use in nursing, an inclusive approach to study selection was maintained. As noted by Hannes and Lockwood [23], qualitative evidence can simultaneously illuminate both broad patterns and context-specific detail, and excluding studies entirely based on quality grounds risks narrowing this breadth. Including all studies regardless of MMAT scores allowed for a representative synthesis across diverse clinical contexts. Quality appraisal results were therefore used to inform the interpretation of findings, rather than as a basis for exclusion. Where lower-quality studies contributed to a construct, this is noted within the relevant Results section.

### Synthesis Approach

Thematic analysis was conducted using a predefined deductive framework based on TAM2, supplemented by the single UTAUT construct of Facilitating Conditions. All included studies were read in full, and relevant text segments, including direct participant quotations, authors’ qualitative summaries, and quantitative findings, were coded line-by-line into the framework constructs. Coding was undertaken by the first author (AJSS), with regular review and discussion of coding decisions with 2 supervising coauthors (BCB and DD) throughout the analysis period. These supervisory discussions served as a calibration mechanism for the application of the codebook; ambiguous extracts and decisions about construct boundaries were brought to these meetings, discussed against operational definitions, and resolved by consensus before being applied across the dataset. The codebook (Table 1) was iteratively refined through these discussions to clarify operational boundaries; construct categories themselves remained fixed throughout. Formal interrater agreement was not calculated, as only a single author (AJSS) coded the data; this is acknowledged as a limitation. Quantitative findings relevant to TAM2 constructs, such as survey results, were summarized and integrated narratively with the qualitative data under relevant headings. Where frequency of reporting is described, this refers to the number of included studies in which relevant data were coded to a given construct. Frequency counts indicate how commonly a construct appeared across the included studies and should not be interpreted as a measure of the depth or strength of any individual theme.

**Table 1.** Codebook adapted from TAM2^a^+UTAUT^b^.

Construct	Definition (as applied in this review)
Perceived usefulness	Excerpts where the AI^c^ is seen as having an impact on job performance. Statements indicating that the AI impacts the effectiveness of task completion or enables more task opportunities. Examples of how AI is perceived to enhance patient care or nursing outcomes.
Perceived ease of use	Descriptions of how the AI is straightforward and easy to use or interact with. Quotes about the learning curves in relation to using AI in practice. Facilitators or challenges related to technical aspects of interacting with the AI.
Subjective norms	Comments about how the organization, management, or colleagues influence attitudes toward AI. Encouragement or pressure from peers to use or not use AI. The role of policy supporting or discouraging AI use.
Intention to use	Nurses’ statements related to plans or indications to use AI in the future. Motivating or deterring factors relating to the intention to use AI in practice. Conditional intentions (eg, willingness to use AI if specific supports or changes are implemented).
Usage behavior	Discussions of actual use or engagement with AI in practice. Context, duration, and frequency of AI use in practice. Examples of tasks or procedures where AI is being implemented.
Facilitating conditions^d^	Technical or organizational infrastructure supporting the use of AI. Availability or lack of resources like support staff, training, and technical expertise. Compatibility of the AI with existing systems and workflows.
Job relevance	To what degree do nurses think AI is relevant to their job. Specific responsibilities or tasks that are impacted by AI. Changes in responsibilities or job roles as a result of AI integration.
Output quality	Perceptions of quality of the results provided by AI systems. AI’s impact on the overall quality, timeliness, and accuracy of nursing care. Consistency and reliability of AI outputs.
Result demonstrability	Visibility and tangibility of results of using AI in nursing practice. Examples of observable and clearly articulated benefits or drawbacks of AI use. Stories demonstrating the impact of AI use on patient outcomes or nursing practice.

a TAM2: Technology Acceptance Model 2.

b UTAUT: unified theory of acceptance and use of technology.

c AI: artificial intelligence.

d Added from UTAUT.

## Results

### Study Selection

**Figure 1.**
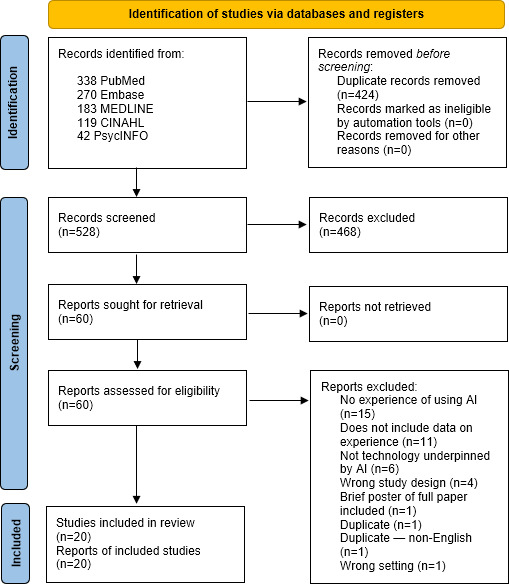
PRISMA (Preferred Reporting Items for Systematic Reviews and Meta-Analyses) flow diagram of study selection.

The PRISMA flow diagram (Figure 1) is presented to illustrate the screening and selection process. The initial searches yielded 952 records. After 424 duplicate or merged records were removed in Covidence, 528 articles were screened by title and abstract. Of these, 60 reports were sought for retrieval and assessed for eligibility, resulting in the exclusion of 40 reports for reasons such as the lack of AI experience (n=15), lack of experiential data (n=11), a focus on non-AI technology (n=6), and other contextual or methodological reasons as detailed in Multimedia Appendix 3. Ultimately, 20 studies [24-43] met the inclusion criteria and were included in the final synthesis.

Of the 20 included studies, 13 were appraised as high quality [31-43], 5 as medium [24-28], and 2 as low [29,30] (Multimedia Appendix 4). The 5 medium-rated studies were predominantly quantitative surveys with limitations relating to sampling representativeness and nonresponse bias [24-28]. The 2 low-rated studies had insufficient methodological reporting to fully assess rigor [29,30]. Job Relevance and Output Quality were supported exclusively or predominantly by high-rated studies. Perceived Usefulness was well-represented across quality levels but anchored by a majority of high-rated studies. Facilitating Conditions drew contributions from the broadest quality range, including both low-rated studies; findings for this construct are therefore interpreted with appropriate caution below.

Of the 20 included studies, the proportion of nurse participants varied substantially. Of the total, 9 studies reported that nurses comprised the majority of participants [24,25,29,31-36]. In the remaining studies, nurses participated alongside other professional groups, and in some cases were a small minority. While there was no requirement for studies to have a majority of nurse participants, this variability affects the extent to which findings directly reflect nursing perspectives. Where possible, data specific to nurses was extracted for synthesis. However, in several studies (eg, [26,34,37]) nursing data were supplementary or aggregated with other professions. A full summary of participant demographics and professional backgrounds is presented in Table 2.

**Table 2.** Characteristics of included studies.

Study (year)	Country and setting	Study design	Participants	AI^a^ and technology focus
Alanzi (2023) [31]	Saudi Arabia, health care sector (government hospitals)	Qualitative, focus group interviews	Total: 54 participants Physicians: 11 Nurses: 24 Dieticians: 8 Pharmacists: 6 Physiotherapists: 5 Sex: 33 females, 21 males Majority have more than 6 years of experience Professions: physicians, nurses, dieticians, pharmacists, and physiotherapists	ChatGPT
Belmin et al (2022) [27]	France, multicenter trial with community-dwelling adults receiving home care	Quantitative, uncontrolled multicenter pragmatic trial	206 community-dwelling older adults Mean age: 85 (SD 8) y Gender: 78% women Age eligibility: ≥75 years Health status: community-dwelling older adults with mild or moderate dependency (GIR 3 or 4) Receiving care from 109 home aides	eHealth system with a machine learning algorithm for predicting ED^b^ visits Decision support for predicting and reducing emergency department visits among older adults, patient monitoring, and optimizing care pathways
Boggiss et al (2023) [38]	Aotearoa, New Zealand, pediatric diabetes clinics and online communities	Qualitative, online focus groups, and one-on-one interviews	Total: 30 Adolescents: 19 (adolescents aged 12-16 y with type 1 diabetes) 58% female, 68% Aotearoa New Zealand European, 11% Māori Living in Aotearoa, New Zealand Diagnosis of type 1 diabetes for more than 6 months No serious developmental or psychiatric disorders Diabetes health care professionals: 11 (health care professionals from various disciplines, such as diabetes nurse specialists, health psychologists, dieticians, and endocrinologists)	COMPASS Chatbot (Expert System) Psychological support and self-management assistance for adolescents with type 1 diabetes
Castagno and Khalifa (2020) [37]	London, United Kingdom, Royal Free London NHS Foundation Trust hospitals	Qualitative, web-based survey	Total: 98 Medical doctor: 34 Nurse: 23 Manager: 11 Therapist: 7 Other: 23 No demographic information was collected	General AI applications in medicine.
Catalina et al (2023) [28]	Central Catalonia, Spain, primary care settings	Quantitative, observational cross-sectional study using a validated survey	Total: 301 Mean age: 46.0 (SD 11.0) year Sex: female (81.1%), male (18.9%) Occupation: Nursing (47.8%), Medicine (48.5%), Others (3.65%) Years of experience: More than 10 years (62.5%)	AI as a health care tool, with a specific focus on its impact on PC radiology.
Gardner and Lundsgaarde (1994) [24]	United States, LDS Hospital	Quantitative, observational study using a questionnaire survey	Total: 1320 Physicians: 360 (aged 27-82 y, mean age 45.5 y) Nurses: 960 (aged 20 to 67 y, mean age 33.57 y) Nurses average professional experience of 9.52 years, with 6.96 years at LDS Hospital	Health Evaluation through Logical Processing (HELP). Expert system Decision support, patient monitoring, and documentation assistance.
Ginestra et al (2019) [29]	Philadelphia, United States, 782-bed academic hospital	Mixed methods, prospective observational study. Surveys were deployed to collect qualitative data on clinicians’ perceptions, and quantitative analysis was performed on the survey results	Total: 287 Nurses: 180 Providers: 107	Early Warning Score tool (EWS 2.0). Machine Learning. Decision Support and patient monitoring for sepsis.
Gonçalves et al (2020) [32]	Brazil	Experience report, direct observation, and semistructured interviews	Not clearly mentioned	Robot Laura. Machine learning. Decision support for identification of sepsis.
Haugsten et al (2023) [39]	Denmark	Qualitative, focus group interviews, and thematic analysis	Total: 14 Doctor: 8 Nurse: 6 Regularly work in a pigmented lesions clinic	ATBM Master (FotoFinder), deep learning, assessment and diagnosis of skin lesions suspicious of melanoma through total body dermoscopy and estimation of malignancy probability.
Helman et al (2022) [33]	Pittsburgh, United States	Qualitative, focus group interviews	Total: 23 Nurses: 14 Nurse Practitioners: 4 Physicians: 5 Median age: approximately 35 years Sex: 60% female Median clinical experience: 8 years	Predictive algorithm for cardiorespiratory insufficiency (CRI) as part of an intelligent decision support system, machine learning, decision support, and patient monitoring.
Im and Chee (2006) [25]	United States	Mixed methods, prospective observational study, and questionnaires	Total: 122 nurses Mean age: 40.26 years Predominantly female (93%) Majority White (75%) 40% have graduate degrees 49% Protestant 59% married	Decision Support Computer Program (DCSP) for cancer pain management. Expert system.
Jauk et al (2021) [34]	Austria	Mixed methods. Convergent parallel design combining questionnaire-based assessments and expert group meetings.	Total: 47 Nurses: 37 Physicians: 10 Sex: male (14), female (33) Age: median 29 (IQR 26‐42) years	Predictive algorithm for delirium management decision support. Machine learning.
Jordan et al (2023) [35]	United States	Qualitative, single-site. Small group and individual semistructured interviews and comparative analysis	Total: 13 ED triage nurses 72.7% female, 27.3% male Age ranges: 45.5% aged 25‐34 years, 27.3% aged 35‐44 years, 18.2% aged 45‐54 years, 9.1% aged 55‐64 years Highest educational degree in nursing: 9.1% nursing diploma, 36.4% associate, 45.5% bachelor, 9.1% master’s Employment status: 9.1% per diem, 27.3% part-time, 63.6% full-time Years of nursing experience: mean 11.5 (SD 10.5, range 1‐35) years	KATE, decision support for emergency nursing triage to improve patient acuity determination. Machine learning.
Koech et al (2022) [40]	Kenya	Qualitative, cross-sectional descriptive, interviews, and focus groups	Total: 70 Focus group discussions: 52 In-depth interviews: 18 Health care workers: diverse group of professionals from antenatal care (ANC) clinic, maternity, outpatient, and radiology departments Community members: pregnant women, partners, and parents Sex: health care workers—7 male, 11 female; Community members—15 male partners, 37 female (pregnant women and mothers) Age groups: health care workers—<35 years (n=7), 35‐44 years (n=6), 45+ years (n=5); Community members—<35 years (n=30), 35‐44 years (n=8), 45+ years (n=14)	TraCer Device, gestational age assessment. Computer vision.
LeBaron and Wang (2023) [41]	United States	Mixed methods, surveys	Total: 40 Clinicians: 5 Nursing students: 19 Medical students: 16	CommSense, real-time feedback to clinicians on communication performance metrics such as medical jargon, interruptions, and speech dominance to improve patient-clinician communication and health care delivery. Natural language processing.
Lintz (2023) [26]	Texas, United States, Rural medical center	Quantitative, cross-sectional questionnaire	Total: 48 Returned questionnaires: 36 Majority younger than 39 years old 70% physicians, 20% registered nurses, 10% other kinds of providers Male: 58%, female: 42%	Hand hygiene monitoring system. Machine learning.
Petitgand et al (2020) [30]	Quebec	Qualitative. Semistructured interviews, informal conversations, nonparticipant observations, with thematic content analysis	Total: 20 DSS developers: 5 AHC managers: 5 Emergency physicians: 7 Nurses: 3	Decision support for diagnostic decision-making in emergency care. Deep learning.
Rui et al (2023) [42]	Massachusetts, United States	Mixed methods. Usability study including observations, think-aloud	Total: 43 Physicians: 21 Pharmacists: 14 Registered nurses: 2 Nurse practitioners: 1 Physician’s assistants: 5 Specialties: internal medicine, neurology, cardiology, oncology or hematology, infectious diseases, endocrinology Average years of practice: 10 years	DynaMed and Micromedex with Watson (DynaMedex). Decision support for clinicians related to drug and disease management. Natural language processing and machine learning.
Sandhu et al (2020) [36]	Duke University Hospital, United States	Qualitative. Semistructured interviews, modified grounded theory approach	Total: 15 ED Physicians: 7 RRT Nurses: 8	Sepsis Watch. Decision support for early sepsis detection and monitoring in emergency departments. Machine learning.
Schwartz et al (2022) [43]	Massachusetts, United States	Qualitative. Semistructured interviews with content analysis	Total: 17 Prescribing providers: 9 Nurses: 8	CONCERN, predictive clinical decision support for in-hospital deterioration.

a AI: artificial intelligence.

b ED: emergency department.

### Quality Appraisal

Using the MMAT [22], adherence to quality criteria ranged from strong methodological coherence [31,33] to studies with notable limitations such as unclear sampling strategies [27], lack of representativeness [26], and insufficient reporting of nonresponse bias [24,25]. Common issues included limited discussion of divergences between qualitative and quantitative findings in mixed methods studies [29,34], and a reliance on single data sources [37]. Detailed MMAT findings for each study are presented in Table S1 in Multimedia Appendix 4.

### Synthesis of Findings

**Figure 2.**
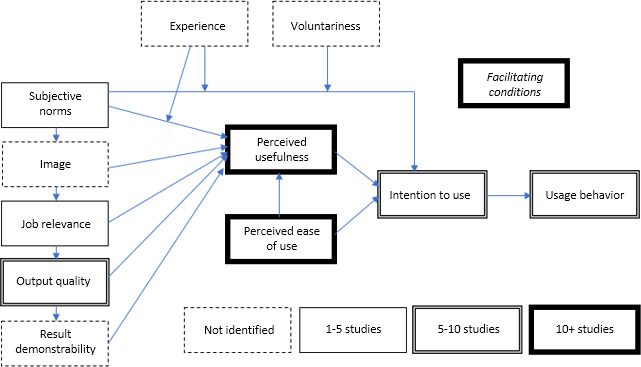
Conceptual map of constructs weighted by frequency.

Findings from the included studies, grouped by TAM2 constructs, are summarized below and presented in detail in Multimedia Appendix 2. Figure 2 illustrates a conceptual map of the TAM2 and UTAUT constructs weighted by frequency of them being identified in the included studies.

#### Perceived Usefulness

A total of 17 studies reported nurses’ perspectives of AI as enhancing the effectiveness and quality of their work [24-27,29,31,33-43]. Reported benefits included earlier detection of clinical deterioration, improved triage and decision-making [29,35,36], more structured and efficient documentation [24,43], and enhanced patient-clinician communication [41]. As 1 nurse explained,

*ChatGPT can help me in learning new developments in the telenursing process, especially in relation to the standards of practices, treatment or monitoring procedures. This helps me to improve my skills and capabilities and be up-to-date with the developments in the telenursing sectors, and provide high quality care to the patients remotely*.

[31]

In some cases, AI tools contributed to professional confidence and visibility [32,35], with nurses describing the technology as a “safety net” in relation to their decision-making [35]. Perceived usefulness was mixed where the tangible benefits to workflow were unclear or where implementation was limited [34,39]. Furthermore, 3 contributing studies [26,34,37] involved multidisciplinary samples from which nurse-specific data could not be disaggregated; these studies are highlighted in Multimedia Appendix 2, and the nurse-attributed findings above are therefore drawn predominantly from the remaining 14 studies.

#### Facilitating Conditions

In total, 16 studies reported on facilitating conditions [24-26,28-31,33,35-41,43]. Common themes included access to adequate training, system interoperability, hardware availability, data privacy, and sufficient time within workflows. Willingness to adopt AI was often expressed as being conditional on meeting these facilitating factors [26,28,40]. For example, 1 nurse noted that,

*Training was yet another organizational matter… not all participants had taken part in these sessions.*

[39]

Nurses also requested integrated feedback mechanisms, such as dashboards and trend data [33,41], which highlights the importance of embedding technical support and continuous learning into implementation strategies.

This construct drew contributions from the broadest quality range of any major theme, including both low-rated studies [29,30] and 4 medium-rated surveys. Petitgand et al [30] additionally only involved 3 nurse participants within a predominantly physician sample. While the consistency of facilitating conditions findings across a large number of high-quality studies lends the construct overall coherence, the specific findings from lower-quality studies should be treated as indicative rather than definitive, and the low-rated studies should be interpreted in the context of their methodological limitations as documented in Multimedia Appendix 4.

#### Perceived Ease of Use

A total of 11 studies addressed perceived ease of use [24-26,28,30,34,36,38,40,42,43]. Nurses valued clear, straightforward interfaces and outputs [36,42], with structured displays facilitating interpretation [24]. As 1 nurse commented,

*The nurses commented that their computer-printed charts were readable and understandable.*

[24]

Barriers included slow system responses, limited interoperability, and difficulty editing records [24,26], as well as challenges interpreting model outputs, with nurses questioning “what have you done to change our processes? What have you done to improve the medical history?” [30]. Several studies noted that ease of use was closely tied to training and resources [28,38,40], which suggests that structured exposure supports both usability and uptake. Moreover, 4 of the contributing studies were medium-rated quantitative surveys, and 1 was rated low [30]; meaning findings on ease of use from these studies are less methodologically robust than those drawn from the high-rated qualitative studies, which provide the primary evidential basis for this construct.

#### Intention to Use

In total, 9 studies described nurses’ intentions to use AI tools [24,27,33,35,39-43]. Intentions were generally reported as positive, with participants expressing interest in continuing or expanding use of AI, particularly with tools that supported decision-making, communication, or workflow efficiency. However, this willingness to adopt was often conditional on receiving adequate training and support, reflecting the critical role of preparatory education in facilitating adoption. All 9 contributing studies reported nurse-specific findings on intention to use, providing a clear nurse-attributable evidence base for this construct; quality ratings of contributing studies were predominantly high (7 high and 2 medium).

#### Use Behavior

In total, 8 studies described nurses’ actual AI system use [24,26,27,29,34-36,43]. Usage ranged from integration into usual practice [36] to limited uptake during pilot testing [34]. Where AI was used, nurses described making changes to patient management and blending system outputs with their own expertise and clinical judgment [27,29]. Lower usage was usually linked to technical limitations, time constraints, or incomplete integration with existing systems. Of the 8 contributing studies, 6 reported nurse-specific use patterns; in 2 studies [26,34], profession-specific use could not be disaggregated and findings reflect aggregated clinician use behavior.

#### Output Quality

A total of 8 studies addressed perceptions of output quality [26,27,31,32,34,35,38,43]. High-quality outputs were described as accurate, relevant, and timely, supporting decision-making and patient care [27,35]. In contrast, neutral or mixed views emerged when outputs were perceived as unclear, lacked contextualization, or when usability issues were present [26,34]. Of the 8 contributing studies, 2 [26,34] involved multidisciplinary samples in which nurses’ perceptions of output quality could not be separated from those of other clinical groups; the findings on output quality therefore reflect a combination of nurse-specific and broader clinician views.

#### Job Relevance

Of the total, 4 studies talked about job relevance [31,32,35,38]. Nurses could see a clear alignment with some core professional tasks, such as maintaining up-to-date clinical knowledge [31], supporting patient self-management [38], aiding early identification of sepsis [32], and enhancing triage decisions without replacing professional nursing expertise [35]. AI systems that aligned with existing nursing responsibilities appeared to reinforce acceptance and perceived value. All 4 contributing studies [31,32,35,38] were appraised as high quality on the MMAT and reported job relevance findings specific to nurses, providing the most consistently nurse-attributable evidence base of any construct in this review, albeit drawn from a small number of studies.

#### Subjective Norms

Of all, 2 studies contained discussion of subjective norms [27,32]. Positive attitudes toward AI among peers and team leaders were associated with greater acceptance, while involvement in the development process contributed to professional satisfaction and perceived status. These findings highlight the influence of workplace culture and peer endorsement in shaping AI adoption. Given that this construct was evidenced by only 2 studies, 1 high-quality qualitative study [32] and 1 medium-quality quantitative study [27], the latter with participant sample limitations including a low response rate, and given the limited evidential base overall, these findings should be treated as preliminary, with low confidence, and interpreted with caution.

Across the included studies, perceived usefulness and facilitating conditions emerged most frequently, highlighting nurses’ focus on whether AI tools tangibly improve patient care and whether the necessary infrastructure and education are in place to support their integration. Perceived ease of use was also a fairly common theme, closely tied to training and ongoing support, suggesting that structured exposure to AI systems is essential for successful adoption. Job relevance and output quality were less frequently discussed but were positive when present, which reinforces the importance of aligning AI applications with core nursing tasks, as well as the need for them to deliver accurate, contextually relevant outputs. In contrast, subjective norms were only sparsely reported, indicating limited exploration of how peer attitudes and organizational culture shape adoption.

## Discussion

### Overview

This systematic review synthesized findings from 20 studies reporting RNs’ experiences of AI in clinical practice. Nurses described AI as having the potential to support decision-making, workflow efficiency, and clinical confidence, although these reported benefits were consistently contingent on implementation quality. Perceived usefulness and facilitating conditions emerged as the most frequently reported constructs, with ease of use, workflow alignment, and organizational support recurring as determinants of whether nurses experienced AI as a benefit or a burden. Findings most directly attributable to nurses come from studies with majority nurse samples (notably for Job Relevance and several studies contributing to Perceived Usefulness), whereas constructs such as Output Quality, Subjective Norms, and elements of Facilitating Conditions drew partially on multidisciplinary samples; these are interpreted with caution below. Confidence in each finding is therefore not uniform; Job Relevance and Output Quality are anchored by high-quality studies, Perceived Usefulness is supported by a mostly high-quality evidence base, Perceived Ease of Use and Facilitating Conditions draw on a wider range of methodological quality, and Subjective Norms rests on the smallest and most heterogeneous evidence base.

### Principal Results

Across the 20 included studies, nurses generally viewed AI as having the potential to improve clinical workflows and to contribute positively to care delivery, although the breadth of these perceptions varied by AI type and clinical setting. However, challenges were also evident, such as training needs, workload concerns, interoperability issues, and ethical considerations. While constructs such as perceived usefulness and facilitating conditions were frequently discussed, subjective norms and job relevance were less often explored, suggesting underresearched areas with potential to influence adoption.

### Comparison With Previous Work

#### Perceived Usefulness

Findings from this review align with the broader AI in health care literature, in which clinicians have expressed optimism about AI’s perceived capacity to support clinical tasks, improve efficiency, and contribute to patient care [44-46]. Nurses in the included studies valued AI for specific applications where they perceived benefits as tangible, such as clinical decision support or rapid information retrieval. However, these perceptions were contingent upon the technology being implemented in a way that integrated meaningfully into existing workflows and practice.

#### Perceived Ease of Use

Consistent with previous research, user-friendly design was a key determinant of willingness to adopt AI in nursing practice [5,47]. Positive experiences were associated with technologies that nurses described as streamlining workflows and reducing burden [48,49]. Conversely, steep learning curves, significant training demands, and inadequate integration led to frustration and resistance [48,50]. Ethical and social concerns, including privacy, trust, and algorithmic bias, were additional barriers [51,52]. These findings suggest that even with positive initial intentions to use AI, without adequate support, integration, and training, this may not translate into long-term adoption.

#### Subjective Norms

Although less frequently addressed in the included studies, subjective norms play a recognized role in technology adoption across contexts [53-55]. Positive cultural attitudes, leadership endorsement, and peer support could foster AI uptake among nurses. Educational settings represent an opportunity to shape these norms early by embedding AI exposure into curricula, normalizing its use, and developing confidence before clinical deployment.

#### Facilitating Conditions

Facilitating conditions emerged as critical to successful implementation. Key barriers included interoperability, data sharing, algorithm transparency, and infrastructure readiness [44]. Education and training were consistently identified as enablers [48,56,57]. Academic institutions can play a pivotal role by equipping future nurses with AI competencies and fostering a culture of informed, confident use. Embedding AI in training environments may help bridge the gap between intention and practice.

#### Job Relevance

While discussed less frequently, job relevance is important for long-term adoption. Concerns included overreliance on AI potentially diminishing nursing roles and the need to ensure AI complements rather than replaces nursing judgment. A proactive approach, where nurses adopt a positive stance toward technological integration [58], may enhance perceived relevance and strengthen the profession’s role in shaping AI use.

### Implications

#### Policy and Practice

Findings from this review reinforce that nurses view AI as a tool to supplement rather than replace clinical decision-making, a perspective echoed in previous research [1,59]. Buchanan et al [59] further call for urgent curricular reform to prepare nurses for AI-enabled health care. Embedding AI into nursing education can demystify the technology, highlight its clinical benefits, and provide hands-on experience that builds confidence and competence.

At a policy level, priorities should include establishing clear interoperability standards, developing robust ethical guidance, and ensuring ongoing professional development for AI use in nursing. At a practice level, health care organizations should actively involve nurses in the design, testing, and evaluation of AI tools to ensure they meet usability and clinical relevance standards. Structured training programs can reduce resistance, improve confidence, and normalize AI use before nurses enter practice, which could help to translate positive perceptions into long-term adoption in real-world clinical settings.

#### Nursing Research

The findings of this review highlight several key areas for future nursing research. First, there is a need for studies exploring the optimal integration of AI systems into nursing workflows, focusing on usability, interoperability, and the impact on patient care outcomes. Research should investigate how AI can supplement clinical decision-making without replacing nursing judgment, which may go some way to addressing concerns about overreliance on technology. Second, given the critical role of education in fostering AI adoption, studies should examine effective strategies for incorporating AI education into nursing curricula and continuing professional development. This includes exploring the use of innovative educational tools to prepare nurses for the evolving health care landscape. Third, research is needed on the long-term effects of AI integration on nursing roles, job satisfaction, and patient-nurse relationships. Finally, studies should address the ethical implications of AI in nursing, including issues of data privacy, algorithmic bias, and the potential transformation of nursing practice. By focusing on these areas, future research can guide the development of AI technologies that enhance nursing practice while preserving the core values of compassionate, patient-centered care.

### Limitations

A key strength of this review is its comprehensive synthesis across diverse AI applications and settings, supported by a transparent search strategy and appraisal process using the MMAT [22]. Another strength is the structured use of the TAM2 framework [16], which provided a consistent lens for analysis and allowed for thematic comparison across studies, although, as noted below, this carries trade-offs for inductive emergence.

There are 6 notable limitations. First, the included studies encompassed a heterogeneous range of AI technologies and contexts, from sepsis prediction tools to natural language processing documentation aids, which limits the generalizability of specific implementation recommendations. In particular, conclusions regarding workflow alignment and facilitating conditions are likely to be highly context-dependent and should not be applied uniformly across all AI tool types or care settings.

Second, many studies involved multidisciplinary participant samples in which nurses were not always the majority, and in several cases, nursing data were aggregated with data from other professions. Where studies included multidisciplinary samples, nurse-specific data were extracted separately where the primary data permitted, as documented in Multimedia Appendix 2. In a small number of studies where profession-specific disaggregation was not possible, findings may reflect broader health care professional perspectives rather than distinctly nursing experiences, and these cases are flagged explicitly in Multimedia Appendix 2.

Third, the deductive analytical framework carries an inherent trade-off. TAM2 was selected a priori and registered with PROSPERO, which ensures transparency and reduces post hoc analytical drift; however, coding line-by-line into prespecified constructs may have constrained the emergence of nursing-specific themes that did not map cleanly onto these categories. We did not encounter substantial extracts that fell entirely outside the framework, although themes relating to professional identity, relational care, or moral distress may be more likely to be visible under inductive analysis. Dimensions such as professional identity, relational care, ethical responsibility, and moral distress in AI-assisted practice may therefore be underrepresented in the synthesis not because they were absent from the primary literature, but because the framework did not create space for them. Inductive or hybrid reanalysis of this literature could potentially surface a complementary picture and is recommended as a priority for future qualitative synthesis.

Fourth, coding of the included studies was undertaken by a single reviewer (AJSS), with regular consultation and consensus discussion of coding decisions with 2 supervising coauthors (BCB and DD) but without independent double-coding of the data. While the consultative process was designed to support consistent application of the codebook, the absence of independent dual coding means that formal interrater agreement could not be calculated, and we cannot fully exclude the possibility of single-coder interpretative bias. This limitation is partially mitigated by the use of a transparent, preregistered framework and by the iterative supervisory review process, but readers should weigh the synthesis findings with this constraint in mind.

Fifth, although the search syntax was not restricted by language, inclusion criteria were limited to studies published in English. This decision was made on practical grounds of interpretive validity, but we acknowledge that it may have excluded relevant empirical work from non-English speaking health care contexts, which could have captured different cultural and organizational dimensions of nurses’ AI experiences.

Finally, the rapid pace of AI development means that nurses’ perceptions are likely to evolve over time, potentially affecting the long-term applicability of these findings.

### Conclusions

This systematic review synthesizes nurses’ experiences with AI in clinical practice, identifying both the opportunities and challenges associated with its integration. While nurses often view AI as a means to enhance workflows, support decision-making, and contribute to patient care, adoption is reported to be hindered by barriers such as usability issues, limited training, and concerns about trust and clinical relevance. It is important to note that this review captures nurses’ reported perceptions and experiences, rather than direct evidence that AI improves patient outcomes, workflow efficiency, or clinical safety; such claims require prospective evaluation. Addressing these challenges requires aligning AI development with user needs and embedding robust education and training into both preregistration curricula and continuing professional development. The consistent findings that facilitating conditions are the primary determinant of positive experience suggest potential value in involving nurses meaningfully in the co-design and iterative refinement of AI tools; this inference is drawn from the weight of evidence on implementation prerequisites rather than direct evidence on participatory design processes, which were only sparsely reported in the included literature. Prospective evaluation using nursing-relevant outcomes, including usability, workflow integration, and trust calibration, is needed to establish which specific implementation conditions reliably produce benefit, and to test whether co-design approaches improve adoption in practice.

By closing these gaps, nursing can play a central role in shaping AI tools that enhance, rather than erode, the human elements of care. As AI capabilities evolve, the profession has an opportunity, and a responsibility, to lead their ethical, effective, and patient-centered implementation.

## Supplementary material

10.2196/91238 Multimedia Appendix 1 Full search strategies.

10.2196/91238 Multimedia Appendix 2 Results summary table.

10.2196/91238 Multimedia Appendix 3 Excluded full-text articles and reasons for exclusion.

10.2196/91238 Multimedia Appendix 4 Quality assessment.

10.2196/91238 Checklist 1 PRISMA checklist.

## Abbreviations

AI: artificial intelligence
JBI: Joanna Briggs Institute
MMAT: Mixed Methods Appraisal Tool
PRISMA: Preferred Reporting Items for Systematic Reviews and Meta-Analyses
RN: registered nurse
TAM2: Technology Acceptance Model 2
UTAUT: unified theory of acceptance and use of technology
