# Supplementary material for: Nurses’ Experiences Using AI in Clinical Practice: Systematic Review
Source: JMIR Nurs. 2026 Jun 25;9:e91238. doi: 10.2196/91238 (PMC13296498; doi:10.2196/91238)
Supplement: Multimedia Appendix 1 [file nursing-v9-e91238-s001.docx]

Multimedia Appendix 1: Full Search Strategies

The following search strategies were used to identify eligible studies for inclusion in this review. Searches were conducted on 13th September 2023 across five electronic databases: PubMed, Embase, MEDLINE, CINAHL, and PsycINFO.

No date restrictions were applied, allowing for the inclusion of studies published at any time up to the search date.

Only studies published in English were included during screening, but language limits were not applied at the search stage.

**CINAHL Complete (EBSCOhost)**

(TI (nurse* OR "nursing staff" OR "nursing professional*" OR "registered nurse*") OR AB (nurse* OR "nursing staff" OR "nursing professional*" OR "registered nurse*"))

AND

(TI ("artificial intelligence" OR "machine learning" OR "neural network*" OR "deep learning" OR "computer vision" OR "natural language processing") OR AB ("artificial intelligence" OR "machine learning" OR "neural network*" OR "deep learning" OR "computer vision" OR "natural language processing"))

AND

(TI (experience* OR perception* OR view* OR attitude* OR feedback OR opinion*) OR AB (experience* OR perception* OR view* OR attitude* OR feedback OR opinion*))

**Embase (OVID)**

(exp Nurses/ or exp Nursing Staff/ or nursing professional*.tw,kf. or registered nurse*.tw,kf.) AND

(exp Artificial Intelligence/ or exp Machine Learning/ or neural network*.tw,kf. or deep learning.tw,kf. or computer vision.tw,kf. or natural language processing.tw,kf.)

AND

(experience* or perception* or view* or attitude* or feedback or opinion*).tw,kf.

**MEDLINE (OVID)**

(exp Nurses/ or exp Nursing Staff/ or nursing professional*.tw,kf. or registered nurse*.tw,kf.) AND

(exp Artificial Intelligence/ or exp Machine Learning/ or neural network*.tw,kf. or deep learning.tw,kf. or computer vision.tw,kf. or natural language processing.tw,kf.)

AND

(experience* or perception* or view* or attitude* or feedback or opinion*).tw,kf.

**PsycInfo (EBSO)**

(TI (nurse* OR "nursing staff" OR "nursing professional*" OR "registered nurse*") OR AB (nurse* OR "nursing staff" OR "nursing professional*" OR "registered nurse*"))

AND

(TI ("artificial intelligence" OR "machine learning" OR "neural network*" OR "deep learning" OR "computer vision" OR "natural language processing") OR AB ("artificial intelligence" OR "machine learning" OR "neural network*" OR "deep learning" OR "computer vision" OR "natural language processing"))

AND

(TI (experience* OR perception* OR view* OR attitude* OR feedback OR opinion*) OR AB (experience* OR perception* OR view* OR attitude* OR feedback OR opinion*))

**PUBMED**

(("nurses"[MeSH Terms] OR "nursing staff"[MeSH Terms] OR "nursing professional*"[Title/Abstract] OR "registered nurse*"[Title/Abstract])

AND

("artificial intelligence"[MeSH Terms] OR "machine learning"[MeSH Terms] OR "neural network*"[Title/Abstract] OR "deep learning"[Title/Abstract] OR "computer vision"[Title/Abstract] OR "natural language processing"[Title/Abstract])

AND (experience*[Title/Abstract] OR perception*[Title/Abstract] OR view*[Title/Abstract] OR attitude*[Title/Abstract] OR feedback[Title/Abstract] OR opinion*[Title/Abstract]))
