# Supplementary material for: Nurses’ Experiences Using AI in Clinical Practice: Systematic Review
Source: JMIR Nurs. 2026 Jun 25;9:e91238. doi: 10.2196/91238 (PMC13296498; doi:10.2196/91238)
Supplement: Multimedia Appendix 3 [file nursing-v9-e91238-s003.docx]

# Multimedia Appendix 3 – Excluded full-text articles and reasons for exclusion.

## Purpose

This appendix lists full-text articles assessed for eligibility but excluded from the review, with the primary reason for exclusion recorded during full-text screening. Within each exclusion category, records are listed in screening order.

## Excluded full-text articles

No experience of using AI (AI discussed conceptually or attitudes/readiness assessed, but no extractable data describing nurses’ direct experience of using AI in clinical practice)

1. Abuzaid MM, Elshami W, Fadden SM. Integration of artificial intelligence into nursing practice. Health Technol (Berl). 2022;12(6):1109-1115. doi:10.1007/s12553-022-00697-0.

2. Ergin E, Karaarslan D, Şahan S, Bingöl Ü. Can artificial intelligence and robotic nurses replace operating room nurses? The quasi-experimental research. J Robot Surg. 2023;17(4):1847-1855. doi:10.1007/s11701-023-01592-0.

3. Glancova A, Do QT, Sanghavi DK, et al. Are We Ready for Video Recognition and Computer Vision in the Intensive Care Unit? A Survey. Appl Clin Inform. 2021;12(1):120-132. doi:10.1055/s-0040-1722614.

4. Laukka E, Hammarén M, Kanste O. Nurse leaders' and digital service developers' perceptions of the future role of artificial intelligence in specialized medical care: An interview study. J Nurs Manag. 2022;30(8):3838-3846. doi:10.1111/jonm.13769.

5. Hamedani Z, Moradi M, Kalroozi F, et al. Evaluation of acceptance, attitude, and knowledge towards artificial intelligence and its application from the point of view of physicians and nurses: A provincial survey study in Iran: A cross-sectional descriptive-analytical study. Health Sci Rep. 2023;6(9):e1543. doi:10.1002/hsr2.1543.

6. King CR, Shambe A, Abraham J. Potential uses of AI for perioperative nursing handoffs: a qualitative study. JAMIA Open. 2023;6(1):ooad015. doi:10.1093/jamiaopen/ooad015.

7. Layard Horsfall H, Palmisciano P, Khan DZ, et al. Attitudes of the Surgical Team Toward Artificial Intelligence in Neurosurgery: International 2-Stage Cross-Sectional Survey. World Neurosurg. 2021;146:e724-e730. doi:10.1016/j.wneu.2020.10.171.

8. Abdullah R, Fakieh B. Health Care Employees' Perceptions of the Use of Artificial Intelligence Applications: Survey Study. J Med Internet Res. 2020;22(5):e17620. doi:10.2196/17620.

9. Swan BA. Assessing the Knowledge and Attitudes of Registered Nurses about Artificial Intelligence in Nursing and Health Care. Nursing Economic$. 2021;39(3):139-143.

10. King CR, Shambe A, Abraham J. Potential uses of AI for perioperative nursing handoffs: a qualitative study. medRxiv. 2022. doi:10.1101/2022.01.08.22268939.

11. Hamer M, Alasmar A, Huber KF, De Camp M. Use and misuse of artificial intelligence-generated prognostication in healthcare settings. J Gen Intern Med. 2023;38(Suppl 2):S303-S304. doi:10.1007/s11606-023-08226-z.

12. Nitiema P. Artificial Intelligence in Medicine: Text Mining of Health Care Workers' Opinions. J Med Internet Res. 2023;25:e41138. doi:10.2196/41138.

13. Van De Sande D, Van Genderen M, Braaf H, et al. Attitude towards artificial intelligence of intensive care unit professionals and their perceived barriers for implementation: A survey study. Intensive Care Med Exp. 2022;10(Suppl 29). doi:10.1186/s40635-022-00468-1.

14. Coronel E, Barringer D, Ross WA, Shafi M, Ge PS. Physician and staff attitudes towards implementation of artificial intelligence-assisted colonoscopy. Gastroenterology. 2022;162(7 Suppl):S-841. (Conference abstract).

15. Strauss AT, Sidoti CN, Jain VS, et al. Artificial intelligence: The newest member of the liver transplant evaluation team? Hepatology. 2021;74(Suppl 1):370A. doi:10.1002/hep.32188.

### Wrong study design (did not meet inclusion criteria for primary empirical studies of nurses’ experiences; eg, systematic/scoping review, editorial/perspective, commentary)

16. Raymond L, Castonguay A, Doyon O, Paré G. Nurse practitioners' involvement and experience with AI-based health technologies: A systematic review. Appl Nurs Res. 2022;66:151604. doi:10.1016/j.apnr.2022.151604.

17. Choi J, Woo S, Ferrell A. Artificial intelligence assisted telehealth for nursing: A scoping review. J Telemed Telecare. 2023;1357633X231167613. doi:10.1177/1357633X231167613.

18. Wilson RL, Higgins O, Atem J, et al. Artificial intelligence: An eye cast towards the mental health nursing horizon. Int J Ment Health Nurs. 2023;32(3):938-944. doi:10.1111/inm.13121.

19. Doria AS. Applications of artificial intelligence in clinical management, research, and health administration: imaging perspectives with a focus on hemophilia. Expert Rev Hematol. 2023;16(6):391-405. doi:10.1080/17474086.2023.2192474.

### Wrong setting (population/setting outside scope; eg, student nurses rather than nurses in clinical practice)

20. Labrague LJ, Aguilar-Rosales R, Yboa BC, Sabio JB. Factors influencing student nurses' readiness to adopt artificial intelligence (AI) in their studies and their perceived barriers to accessing AI technology: A cross-sectional study. Nurse Educ Today. 2023;130:105945. doi:10.1016/j.nedt.2023.105945.

Does not include data on experience (AI/technology described or implemented, but no extractable data on nurses’ experiences of using AI in clinical practice)

21. Griner TE, Thompson M, High H, Buckles J. Artificial Intelligence Forecasting Census and Supporting Early Decisions. Nurs Adm Q. 2020;44(4):316-328. doi:10.1097/NAQ.0000000000000436.

22. Woodnutt S, Allen C, Snowden J, et al. Could artificial intelligence write mental health nursing care plans? J Psychiatr Ment Health Nurs. 2023. doi:10.1111/jpm.12965.

23. Yoo HJ, Lee H. Critical role of information and communication technology in nursing during the COVID-19 pandemic: A qualitative study. J Nurs Manag. 2022;30(8):3677-3685. doi:10.1111/jonm.13880.

24. Junttila K, Meretoja R, Seppälä A, et al. Data warehouse approach to nursing management. J Nurs Manag. 2007;15(2):155-161. doi:10.1111/j.1365-2834.2007.00690.x.

25. Schario ME, Bahner CA, Widenhofer TV, et al. Chatbot-Assisted Care Management. Prof Case Manag. 2022;27(1):19-25. doi:10.1097/NCM.0000000000000504.

26. Winterbottom F. Focus on inpatient sepsis: artificial intelligence, clinical decision support, and e-rapid response. Crit Care Med. 2023;51(1 Suppl):610. doi:10.1097/01.ccm.0000910632.71222.dd.

27. Yu AF, Ferraro E, Liu JE, et al. Home cardiac surveillance with artificial intelligence digital patient monitoring during treatment with pertuzumab, trastuzumab and hyaluronidase-zzxf for HER2-positive breast cancer (HARRIET): study design and rationale. J Am Coll Cardiol. 2022;79(9 Suppl):2004. (Conference abstract).

28. Hong JC, Eclov N, Stephens SJ, et al. Healthcare Staff Sentiment of Clinical Machine Learning Implementation on the Prospective System for High Intensity Evaluation During Radiotherapy (SHIELD-RT) Study. Int J Radiat Oncol Biol Phys. 2020;108(3 Suppl):e780. doi:10.1016/j.ijrobp.2020.07.238.

29. Long A, Lindsey AM, Willetts J, et al. Reducing hospitalizations with artificial intelligence and clinical decision support: lessons learned. J Am Soc Nephrol. 2019;30:1014-1015. (Conference abstract).

30. Ferguson K, Agarawal S. Oral chemotherapy adherence can be improved by oncology nurses utilizing technology. Support Care Cancer. 2019;27(Suppl 1):S90. doi:10.1007/s00520-019-04813-1.

31. Onuigbo M, Agbasi N. SUN-231 The CKD EXPRESS©: the value contribution to chronic kidney disease care using an innovative remote EMR-based monitoring system - a Vermont population health initiative. Kidney Int Rep. 2019;4(7 Suppl):S255-S256. doi:10.1016/j.ekir.2019.05.635.

### Not technology underpinned by AI (technology did not meet the review’s definition of AI; eg, telepresence/telemedicine/robotics/decision support without AI components)

32. Becevic M, Clarke MA, Alnijoumi MM, et al. Robotic Telepresence in a Medical Intensive Care Unit--Clinicians' Perceptions. Perspect Health Inf Manag. 2015;12:1c.

33. Mendez I, Jong M, Keays-White D, Turner G. The use of remote presence for health care delivery in a northern Inuit community: a feasibility study. Int J Circumpolar Health. 2013;72. doi:10.3402/ijch.v72i0.21112.

34. Beran TN, Ramirez-Serrano A, Vanderkooi OG, Kuhn S. Reducing children's pain and distress towards flu vaccinations: a novel and effective application of humanoid robotics. Vaccine. 2013;31(25):2772-2777. doi:10.1016/j.vaccine.2013.03.056.

35. Rogove HJ, McArthur D, Demaerschalk BM, Vespa PM. Barriers to telemedicine: survey of current users in acute care units. Telemed J E Health. 2012;18(1):48-53. doi:10.1089/tmj.2011.0071.

36. Rantanen P, Parkkari T, Leikola S, Airaksinen M, Lyles A. An In-home Advanced Robotic System to Manage Elderly Home-care Patients' Medications: A Pilot Safety and Usability Study. Clin Ther. 2017;39(5):1054-1061. doi:10.1016/j.clinthera.2017.03.020.

37. Tariq A, Westbrook J, Byrne M, Robinson M, Baysari MT. Applying a human factors approach to improve usability of a decision support system in tele-nursing. Collegian. 2017;24(3):227-236. doi:10.1016/j.colegn.2016.02.001.

### Duplicate (duplicate record or duplicate data identified during full-text assessment)

38. Petitgand C, Motulsky A, Denis J-L, Régis C. Investigating the Barriers to Physician Adoption of an Artificial Intelligence-Based Decision Support System in Emergency Care: An Interpretative Qualitative Study. Stud Health Technol Inform. 2020;270:1001-1005. doi:10.3233/SHTI200312. (Duplicate record).

39. Schleder Gonçalves L, de Medeiros Amaro ML, de Lima Miranda Romero A, et al. Implantação de algoritmo de inteligência artificial para detecção da sepse. Rev Bras Enferm. 2020;73(3):1-5. doi:10.1590/0034-7167-2018-0421. (Duplicate record; non-English).

40. Haugsten ER, Vestergaard T, Trettin B. Experiences regarding use and implementation of AI-supported follow-up of atypical moles at a dermatological out-patient clinic; a qualitative study. Acta Derm Venereol. 2022;102(Suppl 222):31. doi:10.2340/actadv.v102.2564. (Brief poster/duplicate data of included full paper).
