# Supplementary material for: Nurses’ Experiences Using AI in Clinical Practice: Systematic Review
Source: JMIR Nurs. 2026 Jun 25;9:e91238. doi: 10.2196/91238 (PMC13296498; doi:10.2196/91238)
Supplement: Multimedia Appendix 4 [file nursing-v9-e91238-s004.docx]

Multimedia Appendix 4 - Mixed-Methods Appraisal Tool (Hong et al., 2018) Quality Assessment

| **Citation** | **Study Design** | **Mixed-Methods Appraisal Tool Summary** | **Rationale** | **Appraisal** |
| --- | --- | --- | --- | --- |
| (Alanzi, 2023) | Qualitative | S1=Y; S2=Y; 1.1=Y; 1.2=Y; 1.3=Y; 1.4=Y; 1.5=Y |  | High |
| (Belmin et al., 2022) | Quantitative | S1=Y; S2=Y; 4.1=?; 4.2=Y; 4.3=Y; 4.4=?; 4.5=Y | 4.1 - Doesn't specify the sampling strategy in detail to determine its relevance to the research question. 4.4 Doesn't provide enough information on the follow-up with participants or the handling of nonresponses to assess the risk of nonresponse bias. | Medium |
| (Boggiss A et al., 2023) | Qualitative | S1=Y; S2=Y; 1.1=Y; 1.2=Y; 1.3=?; 1.4=Y; 1.5=Y | 1.3 - Can't tell because the paper does not include the entire range of qualitative data. | High |
| (Castagno & Khalifa, 2020) | Qualitative | S1=Y; S2=Y; 1.1=Y; 1.2=Y; 1.3=Y; 1.4=Y; 1.5=Y | 1.5 - Survey is sole data source, so no comparator. | High |
| (Catalina et al., 2023) | Quantitative | S1=Y; S2=Y; 4.1=Y; 4.2=?; 4.3=Y; 4.4=?; 4.5=Y | 4.2 - No data on overall demographics of region. 4.4 - No analysis of nonresponse bias. | Medium |
| (Gardner & Lundsgaarde, 1994) | Quantitative | S1=Y; S2=Y; 4.1=Y; 4.2=?; 4.3=Y; 4.4=N; 4.5=Y | 4.2 - Nonprobabilistic sample - unclear if fully representative. 4.4 - There was a significant difference in response rates between physicians and nurses, possibly affecting the overall results. The higher response rate from physicians may be attributed to the non-anonymity of their responses, allowing for follow-up with non-responders​​. | Medium |
| (Ginestra et al., 2019) | Mixed-Methods | S1=Y; S2=Y; 5.1?; 5.2=Y, 5.3=Y; 5.4=?; 5.5=? | 5.1 - no explicit rationale given. 5.4 - Results discusses findings and perceptions, but no discussion of divergence between qual and quant results. 5.5 - Not enough data to decide. | Low |
| (Gonçalves et al., 2020) | Qualitative | S1=Y; S2=Y; 1.1=Y; 1.2=Y; 1.3=Y; 1.4=Y; 1.5=Y |  | High |
| (Haugsten ER et al., 2023) | Qualitative | S1=Y; S2=Y; 1.1=Y; 1.2=Y; 1.3=Y; 1.4=Y; 1.5=Y |  | High |
| (Helman et al., 2022) | Qualitative | S1=Y; S2=Y; 1.1=Y; 1.2=Y; 1.3=Y; 1.4=Y; 1.5=Y |  | High |
| (Im & Chee, 2006) | Quantitative | S1=Y; S2=Y; 4.1=Y; 4.2=?; 4.3=Y; 4.4=?; 4.5=Y | 4.2 - Representativeness not clearly stated. 4.4 - No detailed information on non-response rate or attempts to address non-response bias. Incentives were offered for participation. | Medium |
| (Jauk et al., 2021) | Mixed-Methods | S1=Y; S2=Y; 5.1Y; 5.2=Y, 5.3=Y; 5.4=?; 5.5=Y | 5.4 - Any divergences in data not specifically addressed. | High |
| (Jordan et al., 2023) | Qualitative | S1=Y; S2=Y; 1.1=Y; 1.2=Y; 1.3=Y; 1.4=Y; 1.5=Y |  | High |
| (Koech A et al., 2022) | Qualitative | S1=Y; S2=Y; 1.1=Y; 1.2=Y; 1.3=Y; 1.4=Y; 1.5=Y |  | High |
| (LeBaron et al., 2023) | Mixed-Methods | S1=Y; S2=Y; 5.1Y; 5.2=Y, 5.3=Y; 5.4=?; 5.5=Y | 5.4 - Divergencies and inconsistencies between quant or qual data is not discussed. | High |
| (Lintz, 2023) | Quantitative | S1=Y; S2=Y; 4.1=Y; 4.2=?; 4.3=Y; 4.4=?; 4.5=Y | 4.2 - there is insufficient information to determine if the sample is representative of the broader population of healthcare providers in similar settings. 4.4 - The study achieved a 75% response rate. However, there is limited information on the characteristics of non-respondents to assess the risk of nonresponse bias fully. | Medium |
| (Petitgand C et al., 2020) | Qualitative | S1=Y; S2=Y; 1.1=Y; 1.2=Y; 1.3=?; 1.4=?; 1.5=? | 1.3 - 1.5 - There is not enough data provided in the paper to ascertain that these conditions were met. | Low |
| (Rui A et al., 2023) | Mixed-Methods | S1=Y; S2=Y; 5.1Y; 5.2=Y, 5.3=Y; 5.4=?; 5.5=Y | 5.4 - no discussion of divergencies or inconsistencies in the data. | High |
| (Sandhu et al., 2020) | Qualitative | S1=Y; S2=Y; 1.1=Y; 1.2=Y; 1.3=Y; 1.4=Y; 1.5=Y |  | High |
| (Schwartz et al., 2022) | Qualitative | S1=Y; S2=Y; 1.1=Y; 1.2=Y; 1.3=Y; 1.4=Y; 1.5=Y |  | High |
| Y=Yes; N=No; ?=Can't Tell | | | |  |
